# Musical training heightens auditory brainstem function during sensitive periods in development

**DOI:** 10.3389/fpsyg.2013.00622

**Published:** 2013-09-19

**Authors:** Erika Skoe, Nina Kraus

**Affiliations:** ^1^Auditory Neuroscience Laboratory, Department of Communication Sciences, Northwestern UniversityEvanston, IL, USA; ^2^Department of Neurobiology and Physiology, Department of Otolaryngology, Institute for Neuroscience, Northwestern UniversityEvanston, IL, USA

**Keywords:** development, musical training, auditory brainstem response, sensitive periods, experience-dependent plasticity

## Abstract

Experience has a profound influence on how sound is processed in the brain. Yet little is known about how enriched experiences interact with developmental processes to shape neural processing of sound. We examine this question as part of a large cross-sectional study of auditory brainstem development involving more than 700 participants, 213 of whom were classified as musicians. We hypothesized that experience-dependent processes piggyback on developmental processes, resulting in a waxing-and-waning effect of experience that tracks with the undulating developmental baseline. This hypothesis led to the prediction that experience-dependent plasticity would be amplified during periods when developmental changes are underway (i.e., early and later in life) and that the peak in experience-dependent plasticity would coincide with the developmental apex for each subcomponent of the auditory brainstem response (ABR). Consistent with our predictions, we reveal that musicians have heightened response features at distinctive times in the life span that coincide with periods of developmental change. The effect of musicianship is also quite specific: we find that only select components of auditory brainstem activity are affected, with musicians having heightened function for onset latency, high-frequency phase-locking, and response consistency, and with little effect observed for other measures, including lower-frequency phase-locking and non-stimulus-related activity. By showing that musicianship imparts a neural signature that is especially evident during childhood and old age, our findings reinforce the idea that the nervous system's response to sound is “chiseled” by how a person interacts with his specific auditory environment, with the effect of the environment wielding its greatest influence during certain privileged windows of development.

## Introduction

The auditory brain has an awesome capacity to change through experience. But are there limits to this plasticity throughout development? Are there biological guard rails that place limits on experience-dependent plasticity at some points in life or biological stimulants that promote plasticity at others? In this study, we examine these questions, focusing specifically on the auditory brainstem and what it can reveal about sensitive periods in the auditory brain and its ability to respond to sound.

Except in cases of brain death, the auditory brainstem is always “on” and metabolically active (Sokoloff, 1977; Chandrasekaran and Kraus, 2010). As evidence of this, the auditory brainstem response (ABR) is robust even under general anesthesia, during sleep, and while the participant's attention is directed elsewhere (Smith and Mills, 1989; Skoe and Kraus, 2010; Hairston et al., 2013). These steadfast qualities have made the ABR an invaluable clinical tool in the assessment and diagnosis of hearing- and other auditory-related disorders (Hall, 2007). However, the fact that the ABR changes very little even under deep sleep or anesthesia, has led to a stereotyping of the response, with many researchers and clinicians treating the ABR as merely a reflex that preserves many of the acoustic features of the stimulus. Yet through the analysis of large datasets and more complex stimulus conditions, a different picture has emerged (Galbraith, 2008). With this approach, we have learned that the auditory brainstem captures the physics of the sound (timing, fundamental frequency, harmonics, etc.) as well as the *meaning* (i.e., behavioral significance) attributed to that sound. In fact, recent data from developing, mature, and aging populations demonstrate that brainstem nuclei are refined by active interactions with sound occurring over brief (hours) or long (years) timescales (reviewed in: Krishnan and Gandour, 2009; Kraus and Chandrasekaran, 2010; Bajo and King, 2012; Kraus et al., 2012; Strait and Kraus, 2013). For example, across the lifespan, we observe differences in auditory brainstem function depending on the instrument a person plays or the language or languages a person speaks (Krishnan et al., 2010; Krizman et al., 2012a; Strait et al., 2012a), suggesting that the auditory brainstem's fundamental ability to capture sound is chiseled by idiosyncratic experiences with sound.

Despite ample evidence of experience-dependent plasticity in the auditory brainstem, we have an incomplete picture of how specific auditory experiences influence auditory brainstem development (Kraus and Chandrasekaran, 2010; Jeng et al., 2011; Kraus et al., 2012; Strait and Kraus, 2013). Auditory brainstem nuclei have been long considered to develop precociously, with adult-like function shown to emerge within the first two years of life (Salamy et al., 1975). However, this concept has recently been called into question by evidence that the auditory brainstem continues to develop beyond age 2 (Johnson et al., 2008b; Skoe et al., 2013). This new line of research suggests that the “adult-like” state that occurs around age 2 is only temporary, with each subcomponent of the response exhibiting a unique and more protracted developmental profile. We find generally that the ABR continues to change throughout childhood, ultimately overshooting the adult value, with the developmental inflection point (the point where the curvature of the trajectory changes sign) occurring around ages 5–11. After this inflection point the developmental trajectory “returns” to the adult value then stabilizes. Following this period of stabilization, aging-related changes begin to emerge, around the sixth decade of life. Taken together, these developmental processes manifest in a complex developmental trajectory with four main age-dependent features: (1) a steep initial gradient (~neonatal to age 5), (2) an inflection point (ages ~5 to 14), (3) a period of stabilization where the slope approximates zero (ages ~14 to 50) and (4) a shallow gradient during senescence (ages ~50+).

We theorize that this protracted development of the ABR creates greater opportunities for the sensory environment to influence neural function. We further theorize that the shape and time course of the developmental trajectory is biologically determined with the trajectory providing a baseline on which experience-dependent processes can take root. Because of the undulating nature of the baseline, we posit that the influence of experience will wax and wane as the developmental trajectory changes slope over the life course, with the greatest effects coinciding with times when developmental changes are underway (Bengtsson et al., 2005; Fava et al., 2011). The inflection point may then, we speculate, reflect a “high point” within a sensitive window in development when experience-dependent plasticity is expected to be most pronounced (Kral et al., 2013).

Sensitive periods are restricted windows during development when a particular experience can have a profound and lasting effect on the brain and behavior. Knudsen has argued that sensitive periods are emergent “properties of neural circuits” (Knudsen, 2004), that is that they reflect points in development when a particular neural circuit is in a state of transition and therefore most labile. If the neural circuit receives heightened stimulation during that period of lability, this, we theorize, could exaggerate how the circuit responds during that window which, in turn, could affect how the circuit responds at a later point in time. Kral and colleagues have shown that sensitive windows in auditory cortical development coincide with transitory peaks in synaptic density in the cat (Kral and Eggermont, 2007; Kral and Sharma, 2012; Kral et al., 2013), which is consistent with the idea that sensitive periods reflect times of neural abundance (Jolles and Crone, 2012). Assuming the same holds for the auditory brainstem, then the inflection point in the developmental trajectory may reflect the height of synaptic overshoot, and therefore a critical turning point in the balance between synaptic proliferation and synaptic pruning (Kral and Sharma, 2012; Skoe et al., 2013). Synaptic overshoot has been argued to endow flexibility to the developing auditory system, allowing the system to be protected against sensory deprivation and primed to take advantage of sensory enrichment (Kral and Eggermont, 2007). This led us to ask whether the functional overshoot in auditory brainstem development represents a time of heightened interaction between nature and nurture, i.e., where the interaction between biologically-determined developmental processes and specific auditory experiences is most pronounced. We examine this question in a cross-sectional study of more than 700 participants spanning nearly 8 decades in age, by assessing how enriched auditory experience, resulting from extensive musical practice, affects auditory brainstem development.

Musical training comes in many forms. However, at their core, all pedagogies share the common feature of using music to engage sensory, motor, cognitive, emotional, and social skills. Through repeated practice these skills become more integrated and refined, resulting in a domain general enhancement. In the case of the auditory brainstem, the effects of musical training are not specific to musical stimuli (Musacchia et al., 2007; Lee et al., 2009; Bidelman et al., 2011; Strait et al., 2012a) but emerge in response to other complex sounds including speech and environmental sounds (Parbery-Clark et al., 2009a; Strait et al., 2009b, 2013a). This transfer of learning from one domain to another intimates a sharing of neural resources (Besson et al., 2011; Patel, 2011, in press): musical training fine-tunes how music is represented in the brainstem leading to the enhancement of acoustic features that are common to music and speech. These enhancements emerge as a distinctive neural signature, with musicians having earlier brainstem responses, more consistent responses and more robust amplitudes, especially at the high-frequency end of the response spectrum (reviewed in: Kraus and Chandrasekaran, 2010; Kraus et al., 2012; Strait and Kraus, 2013). Knowing that there is this transference between music and speech, we opted to use a short speech stimulus (40-ms “da” syllable) for the current study. We chose this particular speech stimulus because it is spectrotemporally complex yet short enough to capture many dimensions of the biological response to sound with minimal testing time (~20 min), allowing us to more readily accumulate a large data pool. We have used this stimulus for nearly a decade as part of the standard protocol administered to all study participants and over time we have amassed a large dataset from a wide range of participants, enabling us now to provide the first comprehensive examination of how musicianship affects auditory brainstem function throughout life. It is important to note that although we have repeatedly demonstrated musician enhancements for longer speech stimuli (Wong et al., 2007; Parbery-Clark et al., 2009b, 2012b,c; Strait et al., 2012b, 2013a,b), we have not previously seen differences between “musicians” and “non-musicians” for this exact stimulus and collection protocol (unpublished data). However, previous analyses used small groups of participants within narrow age ranges. By examining the data en masse, from a broader, developmental perspective, we expected that musician effects would emerge as a consequence of increased statistical power. We predicted that the musician neural signature (earlier latencies, more robust high-frequency phase-locking, more consistent responses) would be evident throughout the lifespan but that the signature would be most pronounced during periods of developmental change.

## Materials and methods

All procedures were approved by the Northwestern University Institutional Review Board. Adult participants gave their written informed consent to participate. For infant and child participants, informed consent was obtained from the parent or guardian. Verbal assent was obtained from 3–7 year olds, and written assent was collected from 8–17 year olds using age-appropriate language. All participants were paid for their participation.

**Figure 1:**
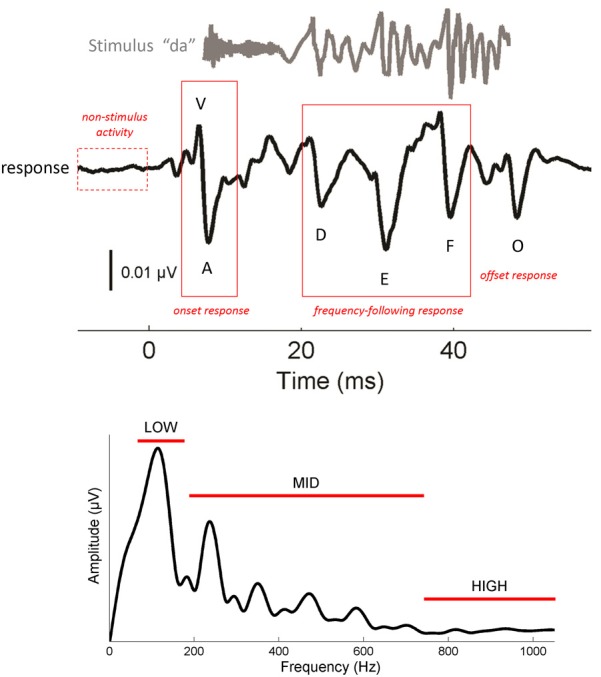
**Characteristics of the cABR (Top).** The complex stimulus [da] (gray) elicits a stereotyped cABR (black) with 6 characteristic peaks (V, A, D, E, F, O). V and A represent the onset response. D, E, and F occur within the frequency-following response (FFR), and O reflects the offset response. The stimulus waveform is shifted by ~6.8 ms to maximize the visual coherence between the two signals in this figure. To obtain a measure of non-stimulus activity, the root-mean-square amplitude of the response to the 15 ms interval preceding the stimulus was taken. **(Bottom)** Frequency domain representation of the FFR (19.5–44.2 ms). Spectral amplitudes were calculated over three frequency ranges: low (75–175), mid (175–750) and high (750–1050 Hz). Waveforms represent the grand averages of the young adult group (21–40 year olds).

Auditory brainstem responses were recorded to a 40-ms speech syllable, /da/, following methodological conventions described previously (Skoe and Kraus, 2010) (Figure 1). We have adopted the terminology “cABR” to refer to ABRs to complex, naturalistic sounds such as speech and music, and will use it to refer to the neural recording throughout this report. cABRs reflect population neural responses from nuclei within the rostral brainstem, including the lateral lemniscus and inferior colliculus (IC) (Chandrasekaran and Kraus, 2010).

### Stimulus

The /da/ stimulus is a five-formant synthesized syllable (Klatt, 1976) consisting of a high-frequency energy burst (occurring at 2500, 3500, and 4000 Hz) during the first 10 ms, followed by a voiced period with a gently ramping fundamental frequency (*F*_0_) (103–125 Hz). During the voiced period, the syllable transitions from a dental place of articulation, characteristic of /d/, to a place of articulation further back in the mouth associated with /a/. This shift in articulation is reflected by linearly changing formant frequencies: the first formant (*F*_1_) ramps up from 220 to 720 Hz, the second formant (*F*_2_) ramps down from 1700 to 1240 Hz, the third formant (*F*_3_) ramps down from 2580 to 2500 Hz, and the fourth and fifth formants are stable at 3500 and 4500 Hz, respectively.

### Parallels between the stimulus and response

One of the most striking features of cABRs is their fidelity to the stimulus (Skoe and Kraus, 2010). As seen in Figure 1, cABRs capture many of the temporal and spectral characteristics of the stimulus. The voiced /da/ stimulus evokes six characteristic response peaks (V, A, D, E, F, O) that relate to major acoustic landmarks in the stimulus, with each peak occurring roughly 6–8 ms after its corresponding stimulus landmark, a timeframe consistent with the neural transmission time between the cochlea and rostral brainstem. (For more information on the neural origins of the cABR we refer the reader to Chandrasekaran and Kraus (2010) where this topic is reviewed). Peaks V and A are transient responses to the energy burst at the onset of the sound, peak O is an offset response that marks the cessation of sound, and the interval spanning D-E-F is the frequency-following response (FFR) to the *F*_0_ of the stimulus and its harmonics. Within the FFR, the interval between the major peaks corresponds to the wavelength of the syllable's *F*_0_. For natural speech, this interval represents the length of each glottal pulse. When air flows from the lungs through the vibrating glottis, a harmonically-rich sound is produced that is then filtered by the speech articulators to give rise to speech formants—concentrations of energy in the speech spectrum. In the cABR, smaller fluctuations between peaks D, E, and F reflect phase-locking to the harmonics of the *F*_0_, up until about 1000 Hz where phase-locking in the IC drops off precipitously (Langner and Schreiner, 1988; Liu et al., 2006). Fourier analysis of the FFR (Figure 1, bottom) reveals spectral peaks at the *F*_0_, and its harmonics, with an amplitude decay at higher-frequencies.

The latency of cABR peaks—the lag between a specific stimulus feature (i.e., onset, offset) and the appearance of a peak—is affected by the stimulus spectrum, including frequencies above 1000 Hz (Johnson et al., 2008a; Skoe et al., 2011). Due to the tonotopic organization of the basilar membrane, higher-frequencies yield slightly earlier peak latencies than lower-frequencies. So while the neural delay between the stimulus and brainstem response falls generally between 6–8 ms, the exact latency is determined by the spectral composition of the stimulus at each particular point in time. Owing to the spectral makeup of the acoustically complex stimulus /da/, energy in the higher end of the spectrum diminishes over the duration of the syllable, such that peak V is being driven more strongly by high-frequencies than the other peaks.

### Participants

The present report includes a total of 770 participants ranging in age 0.25–72.41 years, 213 of whom were categorized as musicians with the remaining representing the “general population” (Table 1). Infants were excluded from the analyses because of the lack of musicians in this age range and also because of the difficulty of defining musicianship in this age; however, they are included for reference in some of the figures. The youngest musician in the sample was 3.26 years old and the oldest was 70.12 years old. Data from many of these musician participants have been previously published for other stimuli. To create the musician group, we pooled data across multiple published and unpublished studies on musicians from our laboratory, adopting the categorization criteria of musicianship for each respective study (Table 2). In large majority, the musicians were “early musicians” (Penhune, 2011), beginning before the age of 7, who practiced on a regular basis.

**Table 1:** **Participant and group characteristics**.

**General population**							**Musicians**						
**Age range**	***N***	**Min.**	**Max.**	**Mean**	**Std. deviation**	**% Females**	**Age range**	***N***	**Min.**	**Max.**	**Mean**	**Std. deviation**	**% Females**
< 1	23	0.26	0.77	0.47	0.14	30.43							
2–5	62	2.44	4.99	4.04	0.66	56.45	2–5	19	3.26	4.98	4.27	0.44	57.89
5–8	26	5.12	7.28	5.80	0.52	69.20	5–8	12	5.12	6.00	5.56	0.34	58.30
8–14	80	8.10	13.73	11.09	1.69	44.30	8–14	15	8.15	13.51	10.68	1.75	60.00
14–17	116	14.00	16.79	14.99	0.60	44.44	14–17	24	14.52	16.32	15.60	0.42	33.33
17–21	44	17.13	21.00	19.59	1.04	54.55	17–21	31	18.09	20.94	19.53	0.76	41.94
21–40	134	21.11	37.36	25.84	4.04	56.72	21–40	68	21.08	38.25	24.92	4.05	45.59
40–60	33	40.30	59.66	51.15	5.87	60.61	40–60	30	45.36	59.66	53.04	3.76	76.67
60–73	39	60.05	72.41	64.13	3.45	74.36	60–73	14	58.83	70.12	62.37	2.55	64.29
Total	557					54.56	Total	213					54.75

Participants were divided into 9 age groups. The number of participants and percentage of female participants is reported along with age statistics (mean, standard deviation, youngest age in group, and oldest age in group).

**Table 2:** **Musician definition by study**.

**Study**	**Age range/mean age (years)**	**Musician definition**			
		**Type**	**Description**	**Mean (years)**	**Age onset (years)**
Strait et al., 2013a,b	3–5	Instrumentalists	12 mo consistent practice, weekly lessons		
Strait et al., 2013a	7–13	Instrumentalists	3+ yrs consistent practice, weekly lessons		≤6
Strait et al., 2012b	7–13	Instrumentalists	Currently undergoing private training, consistently practiced for 4+ yrs (>20 min 5 days+/week)		≤5
*Unpublished*	14–17	Instrumentalists	3+ years of music practice		≤12
Strait et al., 2013a	18–30	Instrumentalists	Continuously practicing with no major gaps, practiced at least 3×/week for 1 h+, received weekly lessons	16.7 ± 3.5	≤7
Wong et al., 2007	18–30	Instrumentalists	6+ yrs continuous training	10.7	≤6
Musacchia et al., 2007	25.6 ± 4.1	Instrumentalists	10+ yrs of musical experience, practiced 3×+/week for 4+ h during the last 10 yrs		≤5
Strait et al., 2009a	19–35	Instrumentalists	2 groups: (1) MusAGE = onset by age 7, (2) MusYRS = 10+ yrs of consistent practice		
Parbery-Clark et al., 2009a	19–30	Instrumentalists	10+ yrs continuous practice		≤7
Lee et al., 2009	25.8	Instrumentalists, vocalists	10+ yrs continuous practice		≤7
Marmel et al., 2011	23.2 ± 4.2	Instrumentalists	11+ yrs continuous contemporary music practice	14.9 ± 5.5	5.7 ± 2
Skoe and Kraus, 2012	18–31	Instrumentalists	6+ yrs music lessons	8.67 ± 1.88	
Strait et al., 2012a	18–35	Instrumentalists	10+ yrs continuous practice leading up to testing time		
Parbery-Clark et al., 2012a	18–65	Instrumentalists	Consistently engaged in musical activities a min of 3×/week “throughout their life”	49	≤9
*Unpublished*	18–40	Instrumentalists, vocalists	6+ yrs music practice		
Parbery-Clark et al., 2012b	45–65	Instrumentalists	Consistently engaged in musical activities min 3×/week “throughout their life”	49	≤9

Age range and musician definition as reported for each respective study.

None of the participants had a history of learning disabilities or neurological dysfunction and all participants had normal audiometric profiles. Normal hearing was confirmed by air-conduction thresholds (<20 dB HL for 500, 1000, 2000, 4000 Hz) for participants older than 5 years or an audiological screen (pass/fail based on distortion product otoacoustic emissions and/or behavioral response at 20 dB HL) for participants 5 and under. To further control for audiometric differences, click-evoked ABRs were recorded on all subjects (Hood, 1998) and confirmed to be within normal limits based on laboratory-internal norms.

Participants were divided into 9 groups by age (<1, 2–5, 5–8, 8–14, 14–17, 17–21, 21–40, 40–60, 60–73 years). Analyses included the 8 oldest groups. Throughout the paper, the age ranges are labeled “X-Y” where X refers to the youngest possible age in the group and Y refers to the next integer value after the maximum age cutoff for the group. For example, for the “2–5” year-old range, 2.00 is the youngest possible age and 4.99 is the oldest possible age. Therefore, there is no overlap between the 2–5 and 5–8 groups.

### Electrophysiological procedures

**Figure 2:**
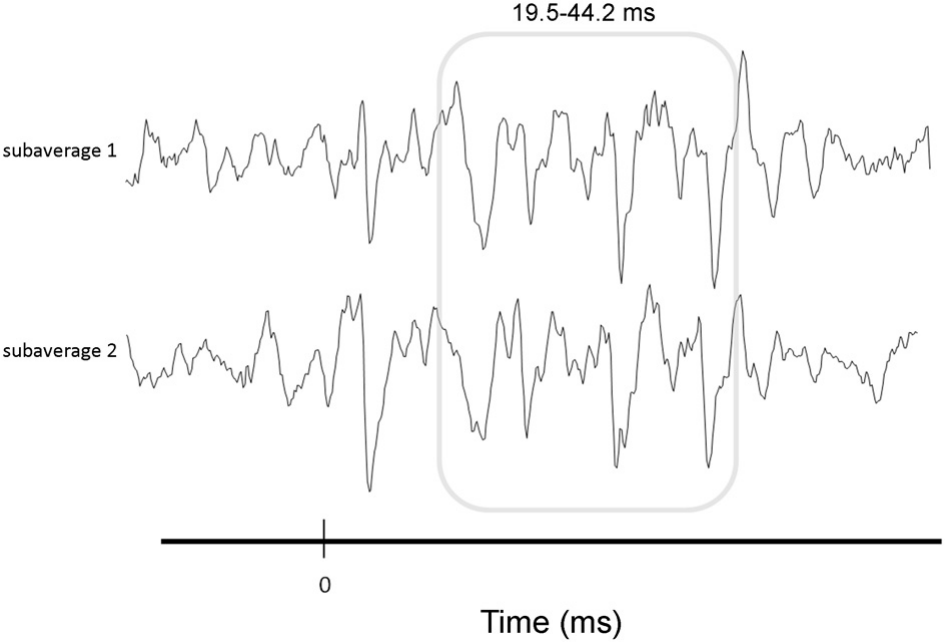
**Illustration of response consistency measure.** To gauge the repeatability of the response over the course of the recording, two subaverages were collected and correlated. Correlations were performed over the FFR (19.5–42.2 ms, gray box).

We briefly summarize the protocol here; for a complete description of the specific protocol we refer the reader to Krizman et al. (2012b). During electrophysiological testing, participants sat in a recliner within a sound treated chamber and were instructed to ignore the stimuli presented to their right ear via insert earphones (10.9/s, 80 dB SPL). cABRs were recorded using the Navigator Pro AEP System (Natus Medical, Inc.). Contact impedance was less than 5 kOhms for all electrodes. A total of 6000 trials were averaged, after excluding trials exceeding +/−23.8 microvolts. To gauge the repeatability of the response over the course of the recoding, two subaverages were collected (Figure 2).

### Analysis

Analysis focused on four sets of measurements: peak latency (6 peaks), FFR amplitude (3 frequency ranges), response consistency, and non-stimulus activity (11 total dependent variables). Latency measurements were made manually via the AEP system, following guidelines described previously (Krizman et al., 2012b). All other data reduction occurred in the MATLAB programming environment (Mathworks, Inc.).

All of the cABR measurements included in the analyses are developmentally sensitive and exhibit age-dependent changes (Johnson et al., 2008b; Skoe et al., 2013). At least two different developmental patterns are expressed in the cABR in typically-developing populations, with the latency and FFR measures displaying a different developmental pattern than the response consistency and non-stimulus activity measures, which have similar but not identical developmental profiles (Skoe et al., 2013). The developmental trajectory for the latency and amplitude measures exhibits a transitory apex during school-age years that briefly overshoots the adult pattern, whereas the other two measures have a more symmetrical, broader trajectory with a more prolonged apex that extends from pre-adolescence into adulthood and lacks an overshoot period (Skoe et al., 2013).

### Peak latency

The response is characterized by 6 peaks, which are highly repeatable within and across participants (Russo et al., 2004; Song et al., 2011). These peaks are referred to as V, A, D, E, F, and O (Figure 1, top). Peak identification was confirmed by a team of experienced observers. Peaks that were not repeatable or did not exceed the noise floor were excluded from the analysis.

### Frequency-following response (FFR) amplitude

The FFR (19.5–44.2 ms) reflects neural discharges that are phaselocked to the *F*_0_ and its harmonics (Moushegian et al., 1973; Skoe and Kraus, 2010). To derive measures of phase-locking at different frequencies, the response was converted to the frequency domain by applying a fast Fourier transform (with zero padding) to the FFR of each participant, after first applying a Hanning ramp. The resultant response spectrum was averaged over three frequency bins: 75–175 Hz (“low”), 175–750 Hz (“mid”), and 750–1050 Hz (“high”) (Figure 1, bottom). The bins were determined based on the acoustic features of the stimulus. The low bin encapsulates the *F*_0_ of the stimulus, the mid bin encapsulates F_1_, and the high bin encapsulates harmonics above the F_1_ that are still within the phase-locking limits of the rostral brainstem (Langner and Schreiner, 1988). The lower and upper boundaries of the analysis bins were set based on visual examination of the morphology of the response spectrum to ensure that the spectral peaks corresponding to the *F*_0_ and *F*_1_ were fully captured in the bin.

### Response consistency

To determine how consistent the FFR was over the course of the recording, we correlated the subaverages using a Pearson product-moment correlation calculation (Figure 2). Values were Fisher transformed to increase the normality of the data prior to analysis (Hornickel and Kraus, 2013).

### Non-stimulus activity

The magnitude of the response in the absence of stimulation was measured by calculating the root-mean-square amplitude of the averaged response to the 15 ms interval preceding the presentation of each stimulus.

### Statistical analyses

To determine how enriched auditory experience affects the developmental trajectory, we conducted an 8 × 2 ANCOVA in SPSS (version 21, IBM) using age group (8 levels) and musician groups (2 levels) as the independent variables for each dependent measure, and covarying for the sex of the participant. For the latency measurements, we also covaried for the click-ABR peak V latency to factor out potential underlying differences in peripheral auditory function between groups (Hood, 1998). *F* and *p*-statistics are reported, along with the Eta squared, the estimated effect size (η^2^). Following Cohen's conventions (Cohen, 1988), an effect size between 0.01 and 0.059 is considered small, between 0.059 and 0.138 is medium, and ≥0.138 is large.

As a planned follow-up analysis, we examined whether the extent of the functional overshoot was larger in musicians compared to the general population. We operationally define *overshoot* to be a point on the developmental trajectory that exceeds the steady-state/stabilization point of the trajectory. To characterize the overshoot, we compared the 5–14 year olds to the young adults, an age range of presumed developmental maturity where the developmental trajectory is relatively stable. By comparing pediatric and adult brains, we adopt a similar approach to the landmark work by Huttenlocher and Dabholkar (1997) who studied synaptic overshoot by examining pediatric and adult human brains post mortem (Huttenlocher and Dabholkar, 1997). For this analysis, we combined the 5–8 and 8–14 year-old groups into a single group for because the 5–14 range appeared to represent a general period of overshoot across the various measures that we examined.

## Results

### Effect of musicianship on the developmental trajectory

**Figure 3:**
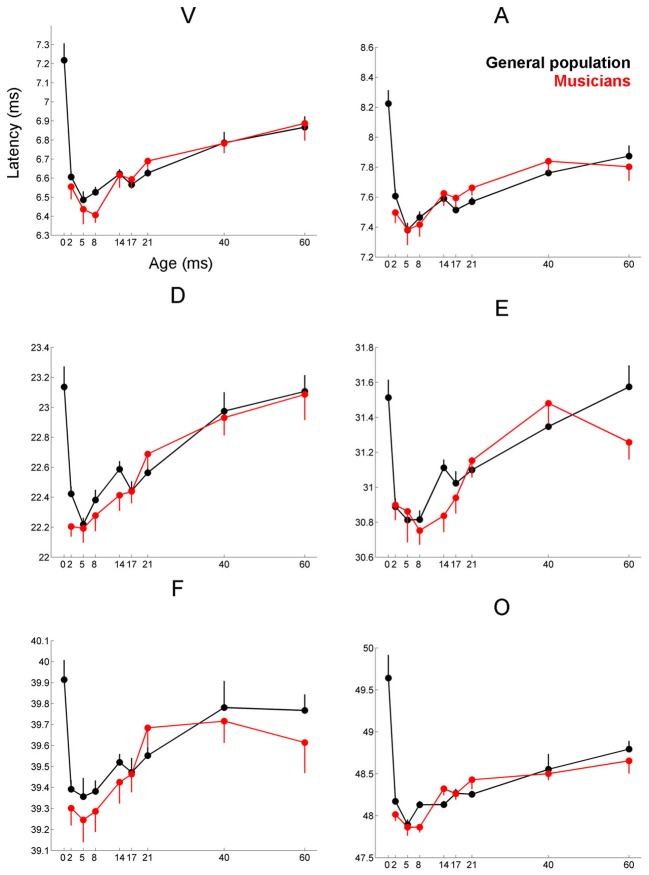
**Age-dependent changes in latency for the six characteristic peaks of the cABR plotted for the musicians (red) and general population (black).** Error bars represent one standard error of the mean (mean + 1 S.E. for the general population and mean − 1 S.E. for the musicians). The value reported on the x-axis represents the youngest age for each group, e.g., 5 represents 5–8 and 8 represents 8–14. The infant group was not factored into the analysis but is plotted here for reference. Across all peaks, the minimum latency occurs around age 8, after this point the latencies progressively delay. We refer to this dip in the latency trajectory as the overshoot; we adopt this term because the latency trajectory approximates the adult value around age 2 and then continues to get earlier for a few years, after which it “returns” to the adult value. For these six peaks, the largest latency differences between the musicians and the general population occur in childhood around the period of the overshoot. Similar patterns are observed across all peaks (except peak A), although only peak V is statistically significant (*p* = 0.035), with E and F showing trending effects.

**Figure 4:**
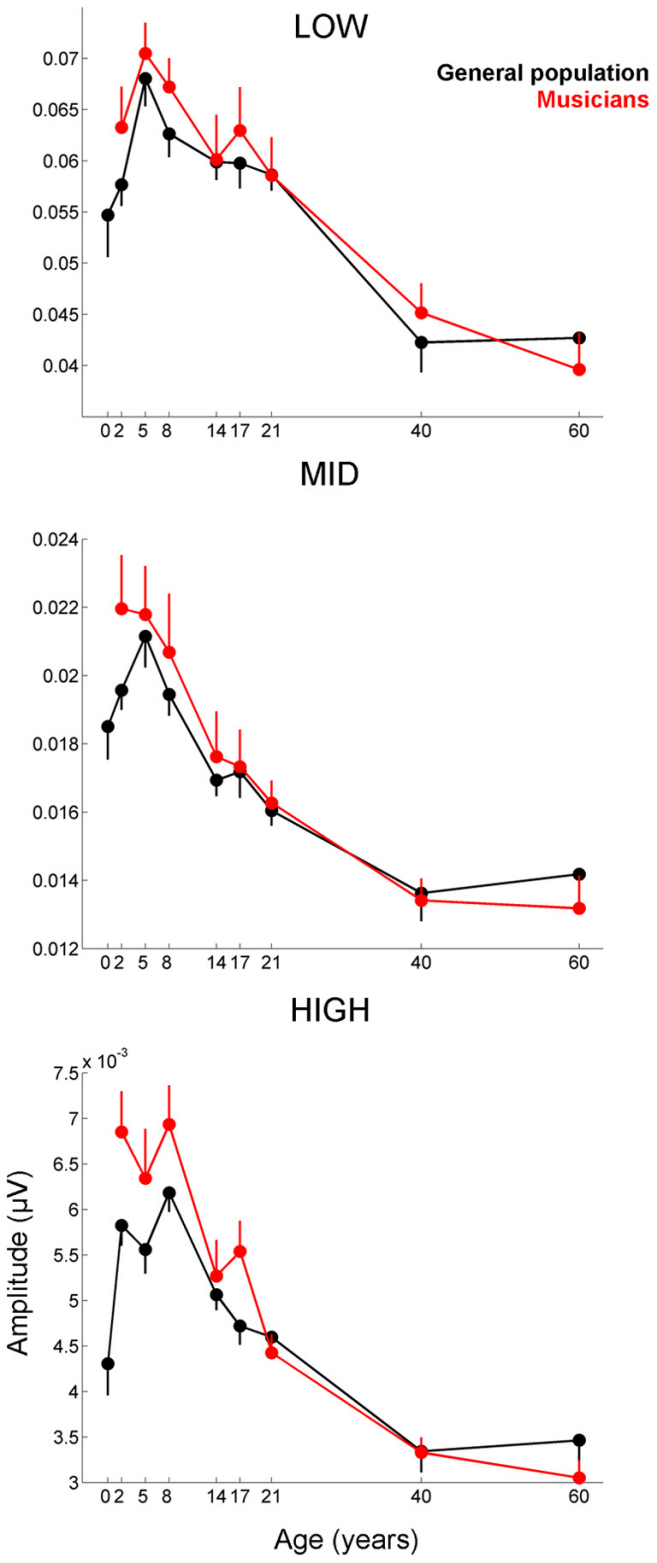
**Developmental trajectories for the low-, mid-, and high-frequency components of the frequency-following response of the cABR are plotted for the musicians (red) and general population (black).** Error bars represent one standard error of the mean (mean − 1 S.E. for the general population and mean + 1 S.E. for the musicians). The value reported on the x-axis represents the youngest age for each group, e.g., 5 represents 5–8 and 8 represents 8–14. The infant group was not factored into the analysis but is plotted here for reference. In all three frequency bands, the response amplitude peaks during early school age years, (i.e., the 5–8 and 8–14 age ranges). A main effect of group was found for the high-frequency region of the response (*p* < 0.014), with the largest differences between the musicians and general population appearing during childhood when the developmental trajectory is cresting. Similar patterns are observed for the other frequency ranges although the statistics are not significant.

**Figure 5:**
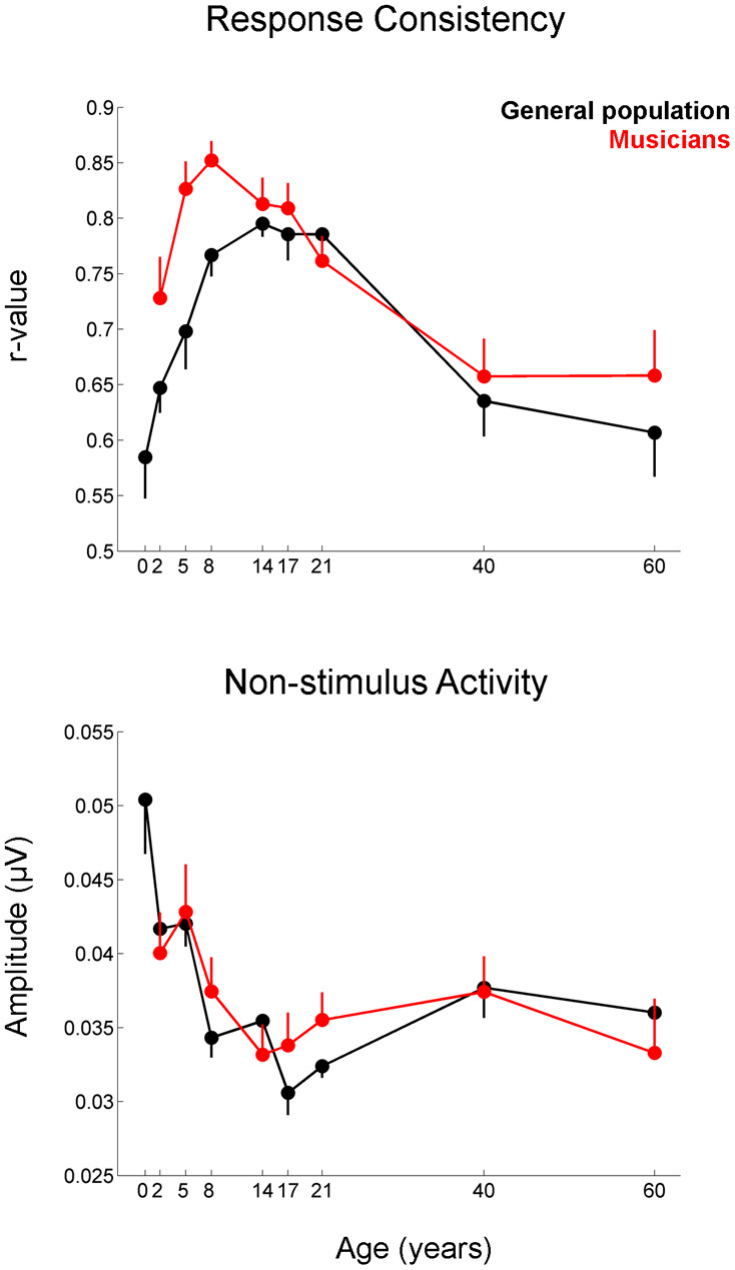
**Developmental trajectories for the measures of response consistency (top) and non-stimulus activity (bottom) of the cABR plotted for the musicians (red) and general population (black).** Error bars represent one standard error of the mean (mean − 1 S.E. for the general population and mean + 1 S.E. for the musicians). The value reported on the x-axis represents the youngest age for each group. Musicians show a distinct trajectory for the response consistency measure (*p* = 0.002) with the differences being most pronounced on the tail ends of the trajectory when the developmental trajectory is most in flux. The groups were matched on the measure of non-stimulus activity.

Musicians were found to differ from the general populations for peak V latency [*F*_(1, 721)_ = 4.469, *p* = 0.035, η^2^ = 0.006], high-frequency phase-locking [*F*_(1, 728)_ = 8.445, *p* = 0.004, η^2^ = 0.011] and response consistency [*F*_(1, 728)_ = 10.742, *p* = 0.001, η^2^ = 0.015]. The main effect of group was trending for E and F latency as was the interaction between age and group for the high-frequency phase-locking measure. No other main effects of group, or group × age interactions were found, (see Table 3 for statistics, Figures 3–5).

**Table 3:**
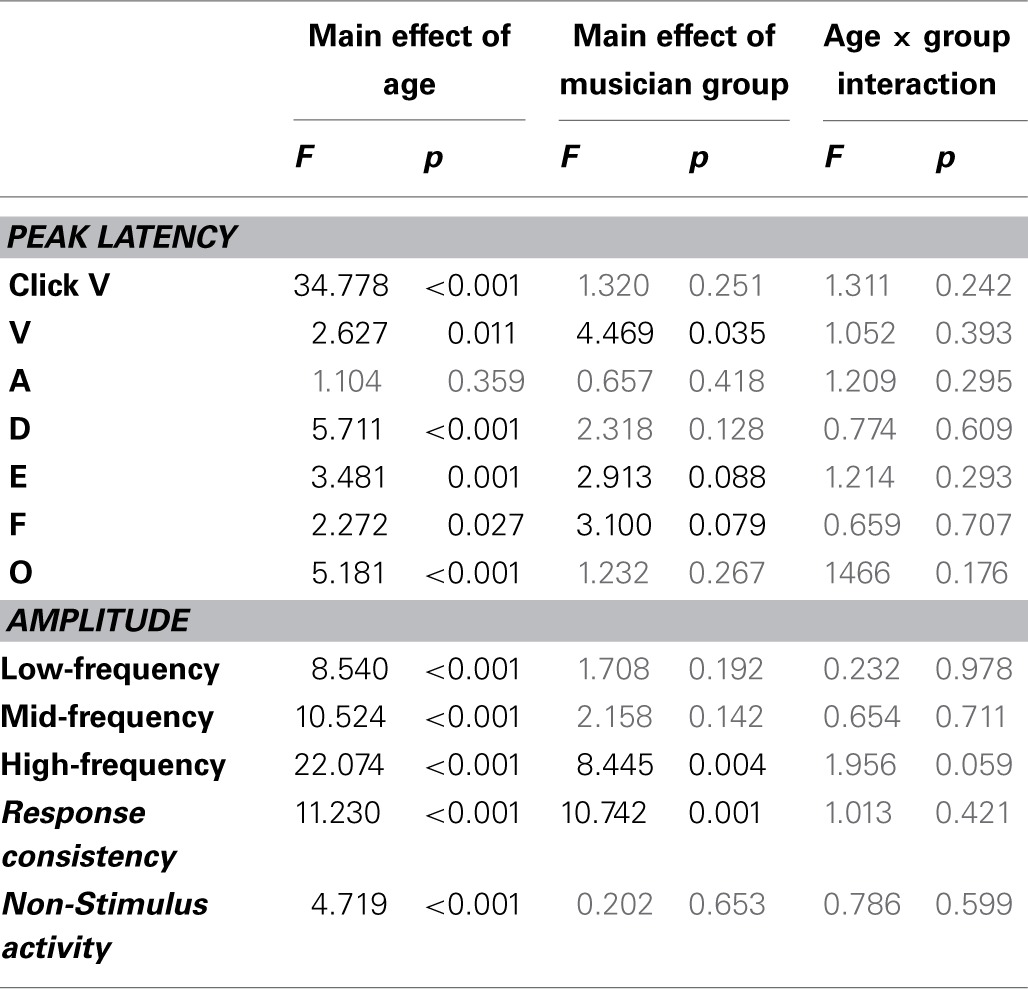
**Summary statistics**.

Omnibus F and p statistics are reported for each independent measure. P-values <0.1 appear in gray. In addition to the 6 peaks of the cABR, results are reported for peak V latency of the click-evoked ABR. The lack of musicianship effects for the click-evoked ABR reinforces that the effects of musicianship on peak V of the cABR are not driven by subclinical differences in peripheral audiometric function.

### Effect of musicianship on the developmental overshoot

**Figure 6:**
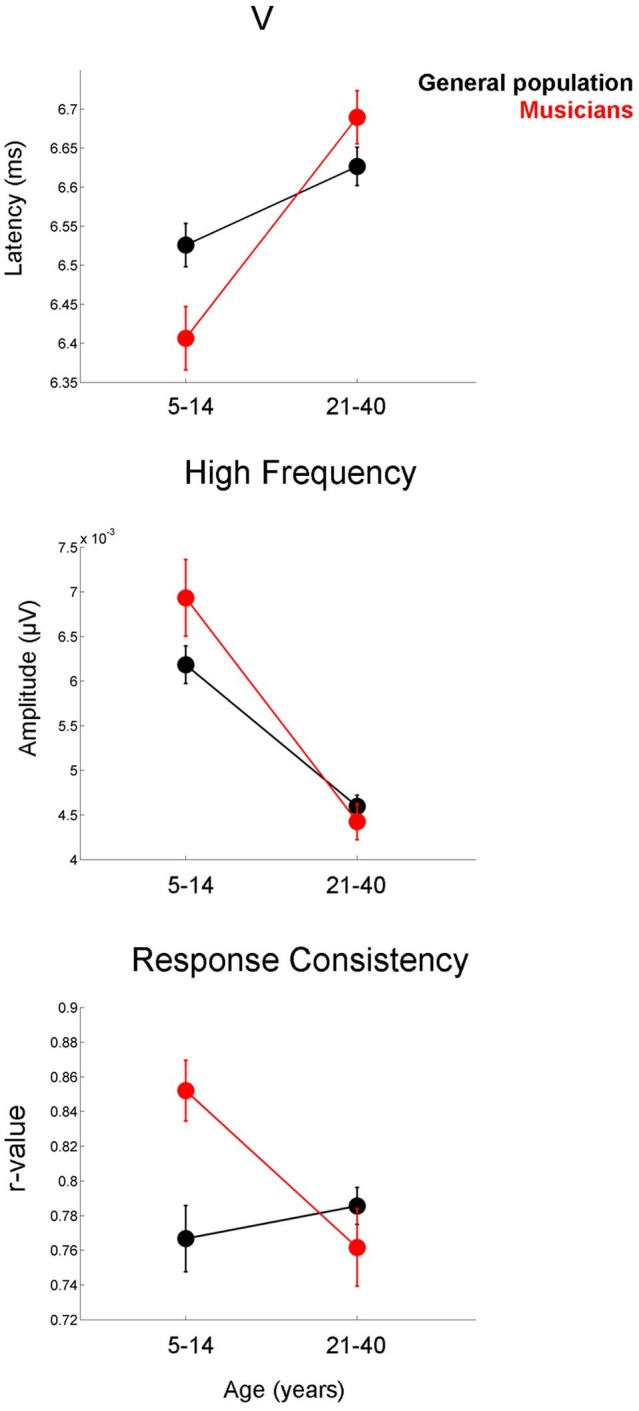
**Musicians (red) display greater developmental overshoot than the general population (black).** This effect is present for the latency of peak V **(top)**, the amplitude of the high-frequency region of the frequency-following response **(middle)**, and response consistency **(bottom)**. Comparisons are made between the 5–14 year-old group and the 21–40 year-old groups. For all three measures, the difference between the child and adult values is greater in the musicians than in the general public. Error bars represent +/− one standard error of the mean.

The developmental profile for the latency and FFR amplitude measures is characterized by a period of overshoot during childhood (occurring within the 5–14 year-old window), when the developmental trajectory briefly surpasses the adult value, as reflected by earlier latencies and larger amplitudes for children of this age compared to the adults (Figures 3–5). The overshoot is observed in musicians and the general population, however, the extent of the overshoot is greater for the musicians for peak V latency and high-frequency phase-locking when comparing the 5–14 year olds to the 21–40 year olds [age × group interaction: *F*_(1, 330)_ = 5.27, *p* = 0.02, η^2^ = 0.016; *F*_(1, 33)_ = 3.23, *p* = 0.05, η^2^ = 0.011, respectively] (Figure 6). For the response consistency measure, the general population shows a u-shaped trajectory, with a prolonged (flat) apex and no overshoot. In contrast, musicians have a less symmetric trajectory for this measure that crests around age 8. Thus, whereas the trajectory is relatively flat for the general population between the child and adult values, musicians show a distinctive developmental pattern in which the musically-trained children have more consistent responses than musically-trained adults. [*F*_(1, 330)_ = 8.53, *p* < 0.005, η^2^ = 0.025] (Figure 6).

## Discussion

Auditory development can be experimentally controlled via deprivation or pharmacological manipulation, leading to the extension, delay, or re-opening of plasticity (Hensch, 2003; McLaughlin et al., 2010; Zhou et al., 2011). This raises the question of whether experiences incurred in the natural world can likewise alter the developmental timeline of the auditory system and manipulate sensitive windows in development (Shahin et al., 2004; Jolles and Crone, 2012). We examined this question by studying the interaction between experience-dependent plasticity and developmental plasticity, using musicians as a model of enriched auditory experience. We aimed to understand (1) which aspects of auditory brainstem development can be altered by musical training and (2) whether there might be windows in life when the effects of enriched experience are most pronounced. We theorized that the potential for experience-dependent plasticity exists throughout life but that experience-dependent processes will “ride” on top of developmental processes resulting in a waxing and waning of experience-dependent plasticity that is constrained by the undulating developmental baseline for each subcomponent of the cABR. Based on previous reports, we also predicted that musical training would not affect all components of the response equally (reviewed in Kraus and Chandrasekaran, 2010; Kraus et al., 2012; Strait and Kraus, 2013). Consistent with our predictions, we observed differences between musicians and the general population for response latency, high-frequency phase-locking, and response consistency—three aspects of the cABR previously shown to be enhanced in musicians (Musacchia et al., 2007; Parbery-Clark et al., 2009a, 2012a,b; Strait et al., 2012b, 2013a,b). Across these different subcomponents of the response, the effect of musicianship appears most evident for younger and older age groups, with minimal differences for the adolescents and young adults (<40 years old). We also observe different developmental peaks and valleys for each component of the musician signature (onset latency, high-frequency phase-locking, response consistency), which lends support to the idea that musician advantages emerge in stages (Strait and Kraus, 2013).

### Music experience maximizes function during sensitive periods in development

The auditory brainstem undergoes at least two different developmental trajectories (Skoe et al., 2013). In the general population, latency and frequency-following components of the cABR have a similar developmental timeline that is marked by a transient period of functional overshoot. Response consistency and non-stimulus activity, on the other hand, exhibit a different developmental timeline that has a more prolonged apex and no overshoot. The current study allowed us to examine whether auditory enrichment, in the form of extensive musical training, alters these developmental profiles.

We find that the general morphology of the latency and frequency-following amplitude developmental trajectories is largely similar between musicians and the general population. In line with the theory that musical-training is constrained by developmental trajectories (Trainor, 2005), this findings suggests that musical training does not speed up or radically alter the shape of the developmental profile for the latency and amplitudes measures. Notably, however, while musical training does not change the *timeline* over which these developmental processes unfold, musical training does appear to interact with these developmental processes. In the case of peak V latency and high-frequency phase-locking, the expression of experience-dependent plasticity is greatest during the period of overshoot. Specifically we found that the functional overshoot is more prominent in musicians for these latency and phase-locking measures, resulting in a bigger difference between the 5–14 year olds (i.e., the height of the overshoot in the general population) and the young adults in the musicians compared to the general population. We take this as evidence that experience-dependent plasticity is maximized during high points in development when neural resources are in abundance and the auditory system is undergoing a sensitive period for these measures.

For response consistency, the morphology of the musician trajectory is, however, rather different from that of the general population. The most notable difference being that the musician trajectory contains an overshoot whereas the general population has a more symmetric profile. Within this qualitatively different looking trajectory, musicians appear to reach developmental high points earlier than the general population, perhaps suggestive of more rapid development of this aspect of the cABR in musicians. The effect can be seen most clearly for the 2–5 year-old musicians whose response consistency is higher than the 5–8 year olds from the general population.

Taken together, in musicians we find that experience-dependent plasticity and developmental processes interact but that the nature of the interaction is different for different subcomponents of the musician signature. In the case of peak V and high-frequency phase-locking, the developmental curves are similar in shape between the musicians and general population, but the musician curve has a more pronounced overshoot. Thus, for these measures, it appears that developmental processes put constraints on how much of an effect the environment can have at each point along the trajectory with a “soft spot” occurring around the period of overshoot, where the effects of musicianship are most amplified. Because the overshoot is not unique to the musician group, we argue that musical training is not triggering the overshoot or controlling the timing of the sensitive period for these subcomponents of auditory brainstem activity. In contrast, for the response consistency measure, musical training seems to change the shape of the developmental trajectory, leading to a period of overshoot that is not evident in the general population. This finding suggests that the environment can trigger changes in developmental processes that underlie the consistency of the response, but that the time points at which this can occur is developmentally constrained.

### Neural mechanisms: changes in synaptic density resulting from active engagement with an enriched environment

Developmental overshoot is thought to reflect a time of neural abundance in which synaptic density is heightened. In the auditory cortex, Kral and colleagues have recently demonstrated that experience-dependent cortical plasticity is increased when auditory experience, which in their case was cochlear implantation, coincides with the point when synpatic overshoot is at its developmental peak (Kral et al., 2013). Analogous to this, our findings suggest that music-related plasticity is heightened during the functional overshoot or in the case of the response consistency measure that musical training triggers an overshoot. We interprets this to mean that enriched auditory experience, in the form of musical training, amplifies neural proliferation in the auditory brainstem [for a related account see (Green et al., 2006)], manifesting in decreased cABR latencies, increased high-frequency amplitudes and increased response consistency relative to the general population during this time period. This early amassing of neural resources, which appears to only be temporary, may protect the nervous system later in life when aging-related processes set in and potentially lead the aging auditory system to operate as if it were biologically younger (e.g., earlier, more robust, and more consistent responses) (Luk et al., 2011; Zendel and Alain, 2011; Parbery-Clark et al., 2012a).

Research from developing and congenitally deaf animals suggests that overshoot in the auditory cortex emerges independent of auditory experience and that the mechanisms leading to the rise, overshoot, and fall of synaptic density are biologically preprogrammed (Kral and Sharma, 2012). We speculate that many of the same general developmental principles hold for the auditory brainstem and auditory cortex, while at the same time acknowledging potential differences between brainstem and cortical development. For example, based on their work with inborn deaf populations, Tillein et al. (2012) have argued that there is no sensitive period in auditory brainstem development (Tillein et al., 2012). In deaf populations, the auditory brainstem (unlike the auditory cortex) remains in a state of arrested development until input is provided after which auditory brainstem development proceeds along a similar trajectory relative to hearing populations no matter when implantation occurs, with the developmental trajectory being driven by “age in sound” instead of biological age (Gordon et al., 2011; Tillein et al., 2012). So while the auditory brainstem has the potential for normal development even if initially completely deprived of auditory input, once auditory input is provided, as is the case for the auditory cortex, developmental processes will ultimately depend on the *nature* and *quality* of that input (whether it be enriched or impoverished) (Moore, 2002; Gordon et al., 2011) as well as how the individual interacts with that input (Kuhl, 2003; Engineer et al., 2004; Kraus and Chandrasekaran, 2010). In addition to receiving an enriched soundscape that supports active and passive music listening, musicians interact with sound in many diverse ways. We believe that it is through the combination of physically producing music, receiving mutlisensory feedback during musical performance, and engaging with music in socially- and emotionally-engaging ways that music is able to affect auditory development and transform how sound is processed by the brain.

### Are these effects specific to musical training?

The functional overshoot for onset latency and high-frequency phase-locking is maximized in musicians. This combined with evidence that musicians exhibit a functional overshoot for response consistency but the general population does not, raises the question of whether we have discovered a sensitive window for musical training in the auditory brainstem? From our perspective, the answer is both yes and no.

We show here that musical training affects specific components of the cABR, which reinforces the concept that musical training produces a selective enhancement and not an overall gain across all components of the cABR. While the specific pattern of enhancements may be unique to musical training, we view functional overshoot as a general property of auditory brainstem development with the time window of the overshoot representing a period of great sensitivity in auditory brainstem development that is not specific to musical training. Thus, the fact that we observed a rather circumscribed effect of musicianship that was limited to a small set of measures does not necessarily mean that the other cABR measures are insensitive to enriched experience or that they lack a sensitive period. Instead we expect that auditory experience of any form, whether it be musical or linguistic, enriched or impoverished, would have an especially pronounced effect during times of developmental change but that different types of auditory experiences might have unique manifestations (reviewed in: Krishnan and Gandour, 2009; Kraus and Chandrasekaran, 2010; Kraus et al., 2012; Strait and Kraus, 2013). For example, musicians have a unique neural signature that can be distinguished from bilinguals (reviewed in Kraus and Nicol, 2014). As we have demonstrated here, musicians tend to have earlier brainstem responses and more robust amplitudes, especially at the high-frequency end of the response spectrum. Boosts in high-frequency phase-locking may reflect a musician's extensive experience with musical timbre, a perceptual feature of sound that is driven (at least in part) by the spectral shape of the harmonics. In contrast, when presented with the exact same stimulus, bilinguals show increased low-frequency phase-locking but no timing enhancements. Increased low-frequency phaselocking may be the outcome of heightened attention to the fundamental frequency, a vocal feature that changes when a bilingual speaker switches languages (Altenberg and Ferrand, 2006; Krizman et al., 2012a). Because bilingualism appears to boost phase-locking to low-frequencies in the cABR but not high-frequencies (Krizman et al., 2012a), we predict that bilinguals will show a distinct developmental trajectory for low-frequency encoding in the auditory brainstem compared to monolinguals.

Thus, we theorize that each cABR subcomponent has the potential to change with auditory experience. We hope to use the current work as a canvas for examining the developmental trajectory of other populations, including bilinguals, to gain a deeper understanding of how developmental processes within the auditory brainstem are influenced by specific auditory experiences.

### Functional significance

Music imparts a specific neural signature on the auditory brainstem. But what is the functional significance of this neural rewiring? For this large dataset, we are not in a position to directly answer this due to the lack of a common behavioral index that can be compared between groups or across ages. There are several reasons for this, with the first being that there is no single behavioral test of perceptual or cognitive function that can be applied to all age groups, from toddlers to older adults. This is in contrast to cABRs, where the exact same testing protocol can be used at all developmental stages. Second, these data were collected over the course of nearly a decade as part of smaller studies where the battery of tests was not entirely overlapping. So, even within an age group we do not have the same behavioral index on all subjects. That said, based on our specific pattern of results and the close mapping between stimulus and the response that characterizes the cABR, we are in a position to speculate on the behavioral significance of our findings. Of the various response peaks, peak V was the most different between the two groups. This peak, which signifies the neural response to the onset of sound, is driven by the initial high-frequency burst of the stop consonant of the stimulus. Earlier onset latencies and more robust high-frequency phase-locking are both indicators of greater neural synchrony in musicians. The combination of earlier latencies and greater high-frequency phaselocking also suggests that musicians might be especially sensitive to the high-frequency, timbral components of the stimulus. This boosting of the higher harmonics, we conjecture, may provide an alternative mechanism for capturing the fundamental frequency of the stimulus given that the harmonics, by definition, are integer multiples of the fundamental.

We also know from previous studies that musicians outperform non-musician peers on a variety of behavioral tasks, including auditory working memory (Parbery-Clark et al., 2009a, 2011a), auditory attention (Strait et al., 2010), and perceiving speech in noise (Parbery-Clark et al., 2009a, 2011a), and that these behavioral advantages correlate with earlier latencies, larger high-frequency responses, and more consistent responses (Parbery-Clark et al., 2009a, 2012b; Kraus et al., 2012). To help further build the case that the neurophysiological differences we observe have functional consequences, our previous work has established that this same set of neural measurements is compromised in children with dyslexia (Wible et al., 2004; Banai et al., 2009; Hornickel et al., 2012). Taken together with our larger body of research, our pattern of findings therefore underscores the idea that the biological processes important for language and cognition are strengthened by musical experience (Patel, 2011; Strait and Kraus, 2011).

### Does more experience translate into more plasticity?

A large majority of our participants were “early musicians” (Penhune et al., 2005) who began music instruction before age 7 and continued to play for many years thereafter. Due to the small sampling of “late musicians” we are unfortunately not in a position to disentangle the effects of when musical training started from how long it lasted; however, given that most of the participants began training around the same age, our dataset can provide insight into how the developmental trajectory changes as more and more experience is accrued.

There are numerous examples indicating that increasing experience accentuates brainstem plasticity (Wong et al., 2007; Strait et al., 2009a; Parbery-Clark et al., 2011b); however, in our cross-sectional survey spanning nearly 8 decades, we find that the musician trajectory does not diverge further from the general population as experience mounts. Instead our findings indicate that musical experience is associated with an initial boost in auditory brainstem function during the first few years of practice, and that additional experience brings about a state of equilibrium in which the differences between musicians and the general population appear diminished when the developmental trajectory stabilizes but then re-emerge later in life when the developmental baseline begins to change. This finding is consistent with evidence that auditory-related plasticity emerges early during learning but “renormalizes” with additional training (Reed et al., 2011) Another, not mutually exclusive interpretation, is that musical training leads the auditory system to operate at its maximal biological capacity at each point in life, allowing the individual to achieve his/her genetic potential for a particular stimulus (Jolles and Crone, 2012). However, once the biological ceiling is met, additional plasticity cannot occur, even if more experience is amassed, unless, for example, there is a change in the underlying biology such as occurs through natural aging.

### When does experience-dependent brainstem plasticity first emerge?

Although experience-dependent plasticity may be maximized during the period of overshoot, our data suggest that experience-dependent plasticity is not limited to this time period. Experience-dependent brainstem plasticity is apparent in the 2–5 year olds, the youngest group of musicians we sampled, as well as the 60–73 year olds, the oldest group of musicians we sampled, with the caveat, however, that we cannot entirely rule out inherent, “baseline” differences between the musicians in our sample and those in the general population. But if our theory holds and experience-dependent effects undulate with age-dependent effects, then given the rapid developmental changes that occur prior to age 2, we predict that music-dependent plasticity could emerge earlier in the rare individuals who begin participating in musical activities before age 2. In the future, we hope to explore this prediction and to also study more generally how early auditory experiences including formal and informal language and music activities (Fava et al., 2011; Trainor et al., 2012; Putkinen et al., 2013) affect auditory brainstem development, interact with the neural mechanisms that give rise to sensitive periods, and lead to changes in auditory proclivities that affect auditory function later in life (McMahon et al., 2012; Yang et al., 2012).

### Comparisons, caveats, and generalizations

This study was performed retrospectively on an existing dataset. While this gave us the benefits of a large dataset, and allowed us to examine the effect of musicianship across nearly 8 decades, the retrospective nature also placed limitations on the study. For example, compared with our previous studies, the groups of participants being considered here are more of a “mixed-bag.” To create the musician group, we carefully combed through our data pool to identify individuals with extensive musical training; however, due to the difficulty of establishing a single “musician” definition that applies to all ages, our musician group is not as precisely-defined as our previous work. For the general population, we also left in individuals with a nominal amount of musical training (<5 years). We took this approach because we wanted to understand how the developmental trajectory manifests under “normal” conditions; given that many individuals in the United States have had some small amount of musical training in school, an individual with zero years of music is in fact quite rare (Steinel, 1990), at least in Middle Class and more privileged populations. So, due to how we created this group, we leave open the possibility that the general population could be displaying a lingering effect of past musical experience (Skoe and Kraus, 2012). We acknowledge that the “muddy” nature of the general population, combined with the uneven number of individuals per group, the greater number of females in the musician group, and the use of a short consonant-vowel stimulus, are all caveats that may have diminished group differences and dampened group by age interactions that appear upon visual inspection of the data (Figures 3–5) but do not emerge in the statistics. For example, in Figure 3, peaks E and F appear different between the two groups, but are only teetering on the verge of being statistically different (*p* < 0.1). This leads us to predict that with more homogenous populations, including uniform group definitions, more extensive latency effects would emerge.

Now turning to the question of whether our findings can generalize to other stimuli. With our short speech syllable, we are able to quickly (20-min paradigm) tap into developmental and experience-dependent processes that are common to speech and music processing. While the pattern of findings is expected to be largely similar regardless of the stimulus, greater effect sizes are anticipated for longer, more complex stimuli (Wong et al., 2007; Strait et al., 2009a), especially when those stimuli are presented in background noise (Parbery-Clark et al., 2009a). The diminishment of neurophysiological differences for the adult participants, we believe, can largely be explained by the stimulus. In young adults, we have previously observed neurophysiological differences between musicians and non-musicians for a similar, albeit longer, “da” speech stimulus, but only when that stimulus was masked by noise, not when it was presented alone (Parbery-Clark et al., 2009a). In contrast, for younger and older populations, musician effects have been seen in both noisy and “quiet” conditions (Parbery-Clark et al., 2012b; Strait et al., 2012b, 2013b). Thus, we treat the apparent lack neurophysiological differences between musicians and the general population during adulthood as being reflective of the specific qualities of our stimulus and not as indicative of a lack of behavioral or other neurophysiological differences.

All caveats aside, the fact that we observed even modest differences between the musically-trained and general populations for a stimulus where musician effects have not previously been reported, we believe makes our findings all the more striking.

## Summary and conclusions

This study examined the interaction between auditory development and enriched auditory experience. Our findings suggest that musical training can intensify neural function during sensitive periods in auditory brainstem development leading to enhancements for specific subcomponents of the cABR and not an overall boost in activity.

### Conflict of interest statement

The authors declare that the research was conducted in the absence of any commercial or financial relationships that could be construed as a potential conflict of interest.
